# Editorial: Autoinflammation of the inner ear

**DOI:** 10.3389/fneur.2022.1071382

**Published:** 2022-11-01

**Authors:** Hiroshi Nakanishi, Takayuki Okano, Taku Ito, Byung Yoon Choi, Michael Hoa

**Affiliations:** ^1^Department of Otorhinolaryngology/Head and Neck Surgery, Hamamatsu University School of Medicine, Hamamatsu, Japan; ^2^Department of Otolaryngology-Head and Neck Surgery, Graduate School of Medicine, Kyoto University, Kyoto, Japan; ^3^Department of Otorhinolaryngology, Tokyo Medical and Dental University, Tokyo, Japan; ^4^Department of Otorhinolaryngology, Seoul National University Bundang Hospital, Seongnam-si, South Korea; ^5^Auditory Development and Restoration Program, NIDCD Otolaryngology Surgeon-Scientist Program, Division of Intramural Research, National Institute on Deafness and Other Communication Disorders, National Institutes of Health, Bethesda, MD, United States

**Keywords:** macrophage, hearing loss, cochlea, *Slc26a4*, *Nlrp3*, corticosteroid

The inner ear has previously been thought of as an immune-privileged organ owing to the existence of its tight junction-based blood-labyrinth barrier. Evidence has revealed that macrophage-like cells exist in the cochlea, and that innate immune responses may cause inner ear inflammation, resulting in sensorineural hearing loss. This Research Topic gathers articles that improve our understanding of tissue-resident macrophages in the cochlea, the association of the innate immune response with sensorineural hearing loss, and prognostic markers for sensorineural hearing loss.

The first article on this topic by Miwa and Okano introduces tissue-resident macrophages in the inner ear. The authors described the origin and diversification of tissue-resident macrophages in the inner ear, as well as in other organ tissues, including the brain, skin, kidney, and spleen. They then discuss the function of tissue-resident macrophages in several systemic autoimmune diseases, such as multiple sclerosis, systemic lupus erythematosus, autoimmune uveitis, Muckle Wells syndrome, and inflammatory bowel diseases. Based on these findings, they speculated on the function of tissue-resident macrophages in hearing loss caused by autoimmune inner ear disease or autoinflammatory diseases.

Liu et al. presented the distribution of immune cells, including macrophages, in human cochlear tissues using immunohistochemistry, confocal microscopy, and multichannel super-resolution structured-illumination microscopy. Multitude of IBA1 positive macrophages scatter in human cochlear tissues, including the spiral ligament, stria vascularis, tympanic covering layer, Reissner's membrane, spiral limbus, and osseous spiral lamina. Cochlear macrophages exhibit different morphological and immunostaining characteristics, depending on their localization. They speculated on the function of macrophages based on their morphological and immunostaining characteristics, such that amoeboid macrophages surrounding spiral ganglion neurons have rich expression of fractalkine genes, suggesting their essential role in nerve protection.

The unique characteristics of tissue-resident macrophages in the stria vascularis have been demonstrated by Ito et al.. A unique subset of tissue-resident macrophages is primarily located adjacent to the blood vessels in the intermediate cell layer of the stria vascularis. Perivascular macrophages form the blood-labyrinth barrier and regulate blood vessel permeability in the stria vascularis, which has been shown to be important for the formation of endocochlear potential. Perivascular macrophages have phagocytic features. They are activated in a mouse model of *Slc26a4* associated hearing loss, resulting in an increase of pigment granules in the cytoplasm and an elevation of hearing thresholds.

Inflammasomes are innate immune sensors expressed in immune cells, including monocytes and macrophages. Of the inflammasomes, the NLRP3 inflammasome is the most well characterized. Recent studies revealed that variants of *NLRP3* cause genetic diseases, including cryopyrin-associated periodic syndrome (CAPS) and non-syndromic sensorineural hearing loss (DFNA34). Patients with both CAPS and DFNB34 present with sensorineural hearing loss characterized by cochlear inflammation caused by *NLRP3* variants. An article by Nakanishi et al. summarizes the current understanding of the mechanisms of the NLRP3 inflammasome, characteristics of patients with CAPS and DFNA34, and the possible pathogenesis of hearing loss.

Previously, no mouse model could be used to investigate the pathogenesis of hearing loss caused by *Nlrp3* variants, since they could not survive to an age when hearing evaluation was possible. Kim et al. reported the first mouse model that manifested quantifiable hearing loss caused by an *Nlrp3* variant. To express the *Nlrp3* variant (p.D301N) in the inner ear, *Nlrp*3^D301NneoR^ mice were crossed with *Gfi1*^*Cre*^ knock-in mice to establish conditional *Nlrp3* variant expression in cochlear and hematopoietic cells. *Nlrp*3^D301NneoR/+^; *Gfi*1^*Cre*/+^ mice exhibited severe to profound hearing loss, and mutant NLRP3 expression was found in the cochlear tissues, including the spiral prominence, outer sulcus, organ of Corti, inner sulcus, and spiral ganglion neurons.

Sudden sensorineural hearing loss (SSNHL) is one of the most common emergent diseases in otolaryngology clinics and has a significant impact on the quality of life. Although the treatment for SSNHL is controversial, the systemic administration of corticosteroids is the most commonly used initial therapy. After treatment, complete recovery was observed in approximately 40% of the patients with SSNHL. Thus, the treatment of SSNHL remains challenging, and its pathogenesis and prognostic factors are still under investigation. Zheng et al. presents an association between liver function and SSNHL. They found that lower hearing impairment at onset and higher serum albumin levels were strongly associated with better treatment outcomes.

There is heterogeneity in the response of patients with hearing instability to corticosteroid therapy. Nelson et al. aimed to characterize the mechanisms by which corticosteroids exert their effects, and the cell types in which they exert their effects in the inner ear. Using single-cell and single-nucleus transcriptome datasets, they revealed that steroid-responsive genes are localized to specific cell types and various regions in the cochlea, including the stria vascularis, the organ of Corti, and spiral ganglion neurons. The analysis demonstrated that steroid-responsive differentially expressed genes in spiral ganglion neurons were associated with angiogenesis, apoptosis, and cytokine-mediated anti-inflammatory pathways.

We hope that the readers will find that this Research Topic improves our understanding of the role of cochlear inflammation and immunity, and provides a better foundation for the development of potential therapeutic treatments for sensorineural hearing loss.

## Author contributions

All authors listed have made a substantial, direct, and intellectual contribution to the work and approved it for publication.

## Conflict of interest

The authors declare that the research was conducted in the absence of any commercial or financial relationships that could be construed as a potential conflict of interest.

## Publisher's note

All claims expressed in this article are solely those of the authors and do not necessarily represent those of their affiliated organizations, or those of the publisher, the editors and the reviewers. Any product that may be evaluated in this article, or claim that may be made by its manufacturer, is not guaranteed or endorsed by the publisher.

